# Thin Electric Heating Membrane Constructed with a Three-Dimensional Nanofibrillated Cellulose–Graphene–Graphene Oxide System

**DOI:** 10.3390/ma11091727

**Published:** 2018-09-14

**Authors:** Chuang Shao, Zhenyu Zhu, Chuwang Su, Sheng Yang, Quanping Yuan

**Affiliations:** School of Resources, Environment and Materials, Guangxi University, Nanning 530004, China; icshao@163.com (C.S.); zhenyzhu@163.com (Z.Z.); glscw58@163.com (C.S.); yangs0825@163.com (S.Y.)

**Keywords:** nanofibrillated cellulose, graphene, graphene oxide, electric heating membrane, electric heating performance

## Abstract

Nanofibrillated cellulose (NFC) and graphene oxide (GO) with reinforcing and film-forming properties were employed with graphene to develop a novel and thin electric heating membrane with heat dissipation controllability. A negative charge was found on the surface of GO and NFC in aqueous dispersions, which contributed to the homogeneous distribution of the graphene sheets. The membrane had a good laminated structure with three-dimensional interaction between GO and NFC, with embedded graphene sheets. Conductivity was characterized as a function of the amount of graphene, thus giving control over to the heating power by adjusting the ratio of graphene. Subsequent electric heating tests can remove irregularities on the GO and graphene sheet, improving the laminated structure further. The temperature on the surface of the membrane presented an exponential increasing regularity with time. Under the same power density and time, the stabilized temperature rise of membranes was higher when grammage was higher, which was characterized by the linear function of the power density. Low-grammage membranes (1 and 4 g·m^−2^) also exhibited regular and even stabilized temperature rises. The indicated structure and heating performance of the membrane, as well as the variation induced by Joule heating, would drive its applications.

## 1. Introduction

Graphene-based materials such as graphene nanosheets, multilayer graphene nanoplatelets, graphene oxide (GO), and reduced graphene oxide (RGO) are currently of considerable interest in many fields, including composite materials, paints and coatings, flexible electronics, energy generation and storage, sensors and metrology, and bioapplications [1,2,3]. In the field of electric heating, new graphene-based composites have been applied for the heating and thermal analysis of microdevices or micro/nano regions of materials [4], as well as being used in snow melting and deicing devices [5,6,7,8], demisting and defrosting of transparent substrates such as glass [9,10,11], wearable/smart electronics [8], and even indoor heating. One such high-temperature heating device utilized a RGO coating on the surface of a horseshoe-shaped substrate by three-dimensional (3D) printing using aqueous solution [4], which was first reduced at 600 °C for 1 h under argon and then further reduced by input electrical current below 1 A. This 3D heater reached a temperature up to 3000 K and displayed extremely fast rise and descent, with speeds of up to ~20,000 K·second^−1^. It was also reported that there was a good linear relation between voltage and electric current, and a high coincidence degree was observed after 50 tests [4]. In addition, it exhibited stable electric heating performance; the current changed by only 0.1 mA after operation at 4 V for 24 h.

However, this Joule heating effect has more diverse applications than simply heating. When RGO is used for current collection in a lithium battery, after Joule heating up to 2750 K for 1 min by applying a direct current, the sheet resistivity has been demonstrated to drop as low as 0.8 Ω/sq, with conductivity of up to 3112 S·cm^−1^ [12]. In addition, graphene-based composites have been produced that have “reversible superwettability”; they can convert from super-hydrophilic to super-hydrophobic in 1 min under Joule heating from an applied electric current [13].

It is clear that endowing graphene and graphene-based composites with electric heating functions has much promise; however, there are few systematic studies focusing on the electric heating performance. While polymer substrates such as polydimethylsiloxane [6] and epoxy resin [5] were used in the above works, graphene material can be fabricated directly into membranes by printing [4], chemical vapor deposition (CVD) [10], or spin coating [11]. However, membranes or other lamellar materials constituted from graphene or RGO without sufficient polar groups at the surface would deteriorate under heat, load, or deformation, etc. To make up for these deficiencies, nanofibrillated cellulose (NFC) is often added to graphene composites for better heat stability, suppleness [14], and tensile strength [15] in reinforcing materials [16], sensing elements [14], and capacitor electrodes [17]. Besides, some other polymers such as polybenzimidazole with outstanding thermal and mechanical stability had been recently used for graphene functional composite [18]. However, NFC, as an abundant natural polymer, has an excellent capacity for forming self-assembled monolayers due to its high surface area, abundant polar groups, and high length-diameter ratio [19]. These monolayers can be combined with GO by hydrogen bonds to form a 3D network [15], thus forming a favorable structure for the homodispersion of graphene-GO-NFC systems. It can be concluded that NFC facilitates and stabilizes the system’s homodispersion [20,21].

Consequently, a new strategy was developed for fabricating NFC-graphene-GO 3D systems for electric heating membranes. A mixture of NFC and GO was first prepared, which acts as a binder and dispersion stabilizer, before the graphene sheet dispersion was added. In addition, the influence of the ratios among graphene, NFC, and GO on the morphology, structure, conduction, and heating performance are systematically reported in this work, as well as the effect induced from Joule heating. Moreover, the relationship of grammage to conductivity and heating performance will be taken into account, aimed to produce a thinner membrane with suitable power efficiency. This presented work endowing the membrane with a new-type of electric heating function would further broaden the application of NFC–graphene based composite.

## 2. Materials and Methods

### 2.1. Materials

NFC dispersion, concentration 1.07 wt.%, diameter 10–20 nm, length 2–5 μm, was manufactured by Tianjin Woodelfbio Cellulose Co., Ltd., Tianjin, China; GO dispersion, concentration 2 mg·mL^−1^, pH 5–7, specific surface area 1000–1217 m^2^·g^−1^, thickness 0.6–1.0 nm, and a lamellae diameter 0.5–5 μm, was from Suzhou TANFENG Graphene Tech. Co., Ltd., Suzhou, China; graphene, was produced by the CVD method, 1–2 layers, purity > 99 wt.%, and a specific surface area of 1000–1217 m^2^·g^−1^, sheet thickness 0.5–3.0 nm, lamellae diameter 0.5–5 μm, was produced by Suzhou TANFENG Graphene Tech. Co., Ltd., Suzhou, China; copper foil electrodes with the a thickness of 0.03 mm and width 5 mm.

### 2.2. Preparation of the Composite Membrane

**Figure 1:**
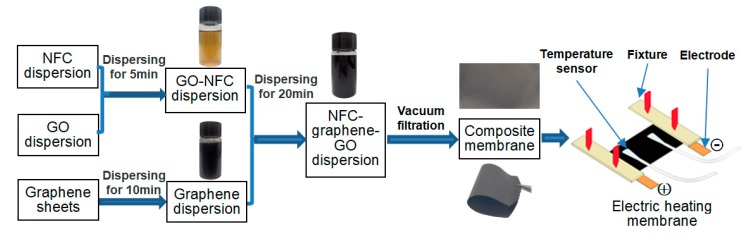
Preparation of the membrane and electric heating device.

The process for producing NFC–graphene–GO composite membranes is summarized in Figure 1. First, a NFC-GO mixture was prepared by mixing a certain proportion of NFC dispersion with GO dispersion, along with 20 mL of distilled water. The mixture was stirred using a magnetic stirrer for 5 min, and then dispersed by ultrasonication (1000 W, 5 min) using an ultrasonic cell crusher (TL-1200Y, Jiangsu Tenlin Instrument Co., Ltd., Yancheng, China).

Next, a graphene dispersion was prepared by mixing a certain amount of graphene with 20 mL of distilled water. The mixture was stirred using a magnetic stirrer for 5 min, and then treated with ultrasonication (1000 W, 10 min).

Finally, a NFC-graphene-GO dispersion was formed by pouring the NFC-GO mixture into the graphene dispersion. The mixture was treated with ultrasonication (800 W, 20 min) and filtered with a 0.22 μm organic filter (Shanghai XINYA Purification Equipment Co., Ltd., Shanghai, China). The dispersion was then placed in a vacuum drying oven at 60 °C for 12 h to obtain NFC-graphene-GO composite membranes, which were kept in an indoor environment (about temperature of 20 °C and humidity of 60%) for 24 h.

Composite membranes were prepared with different grammages or graphene contents, in which the weight ratio of GO to NFC was kept constant, at 1:1. The graphene content was varied from 20 to 55 wt.% in 5 wt.% intervals with a controlled grammage of 16 g·m^−2^ (dry weight); the grammage was varied at 1, 4, 8, 12 and 16 g·m^−2^ with a constant 50 wt.% graphene. Further composite membranes were prepared to investigate the influence of GO on the surface morphology and phase structure. The GO contents were 5, 15, 25, and 30 wt.%, with a constant 50 wt.% graphene, corresponding to GO to NFC weight ratios of GO:NFC = 1:9, 3:7, 1:1, and 3:2.

### 2.3. Electric Heating Membrane and Heating Properties

Composite membranes with different amounts of graphene and grammages were used to prepare electric heating membranes. First, a four-probe resistivity tester (ST2258C, both separation between probes and diameter of probe as 1 mm, Suzhou Jingge Electronic Co., Ltd., Suzhou, China) was used to test the sheet resistance of the membranes at 7 equidistant points (Figure S1, Supplementary Materials), with three repeat measurements for each sample. Then, samples were cut with a size of 20 × 30 mm^2^, to which copper foil electrodes were fixed at both ends, to prepare electric heating membranes with an effective heating surface area of 20 × 30 mm^2^. Electric heating device and installation of electrodes and temperature senor on the membrane can be seen in Figure 1 and Figure S2 (Supplementary Materials).

Change rate of resistance between two electrodes of the membrane after different bending cycles (bending angle of about 90°) of 0–600 times at 100 times intervals were tested for the evaluation on its stability and flexibility using a multimeter (Fluke15B+, Fluke Corp., Washington, DC, USA). The change rate is the percentage of the absolute value of difference between the resistance before and after bending to the resistance before bending.

To improve the stability of the heating performance, membrane annealing was induced by Joule heating [4,12] in a first heating test. A power density of 1500 W·m^−2^ was applied to each membrane for 15 min with a voltage regulator controlling the power, considering the tested resistance between the two electrodes and the effective surface (Calculation for voltage applied on two electrodes is shown in Equation (S1) and Equation (S2), Supplementary Materials). A second heating test was then carried out under the same conditions for specific analysis of the heating performance. In addition, in order to reveal the relation between heating performance and power density, the heating performance of the 8 g·m^−2^ membrane annealed at 1500 W·m^−2^ for 15 min was further analyzed by applying power densities of 500–2500 W·m^−2^ at 500 W·m^−2^ intervals (electrifying time 15 min). In both tests, the temperature was recorded every 2 s at two test points using a multichannel temperature recorder (SIN-R960, Hangzhou SinoMeasure Automation Technology Co., Ltd., Hangzhou, China); the room temperature was simultaneously recorded to calculate the temperature rise (difference between real-time membrane temperature and ambient temperature). The resistance between the two electrodes was recorded before the heating test, at the outage moment, and when cooling to room temperature, to investigate the membrane’s power stability.

### 2.4. Characterization

The morphology of graphene, NFC, and GO dispersions (concentration 0.01 mg·mL^−1^, ultrasonic treatments at 1000 W for 5 min, then 800 W for 20 min), and NFC–graphene–GO dispersion (concentration 0.01 mg·mL^−1^, graphene 50 wt.%, GO:NFC = 1:1 (weight ratio), ultrasonic treatment as for the composite membrane above) were analyzed in an aqueous solution using transmission electron microscopy (TEM; Hitachi HT7700, Hitachi Advanced Microscopy Techniques Corp., Chiyoda-ku, Japan) at 100 kV. Both the surface and cross-section were observed using scanning electron microscopy (SEM; S-3400N, Hitachi, Chiyoda-ku, Japan). The zeta potential and particle size of NFC, GO, and NFC-GO dispersions were analyzed in aqueous solution with the Zetasizer Nano ZSP (Malvern Instruments Ltd., Malvern, UK). The concentrations of the NFC and GO solutions were 0.25 mg·mL^−1^, and that of the mixed dispersion was 0.5 mg·mL^−1^. The 3D surface morphology and roughness (10 × 10 μm area) were analyzed using atomic force microscopy (AFM; Model 5500, Agilent Technologies Inc., Palo Alto, CA, USA) in tapping mode. Fourier transform infrared (FT-IR) spectra of powdered samples were collected from 400 to 4000 cm^−1^ with a Nicolet iS50 spectrometer (Thermo Fisher Scientific, Waltham, MA, USA) that had a resolution better than 0.09 cm^−1^. The phase structure of the membrane was analyzed by laser confocal micro-Raman spectrometry (inVia Reflex, Renishaw, Gloucestershire, UK) from 4000 to 500 cm^−1^ with an excitation wavelength of 532 nm. Each sample was tested three times. X-ray diffraction (XRD; SmartLab 3 kW, Rigaku Corp., Akishima-shi, Japan) was carried out for 2*θ* from 5° to 70° with an 8°·min^−1^ scanning speed on graphene, NFC, GO, and the composite membrane. Differential thermal-thermogravimetric analysis (DTA-TG; DTG-60 (H), Shimadzu Corp., Kyoto, Japan) was applied to study the thermal performance during heating from room temperature to 600 °C at a rate of 10 °C·min^−1^ in a nitrogen atmosphere, with respect to NFC, GO, graphene, and the composite membrane (graphene 20 wt.% and 50 wt.%, GO:NFC = 1:1 (weight ratio)). The surface morphology and phase structure of the membrane (graphene 50 wt.%, GO:NFC = 1:1 (weight ratio)) were evaluated after the two electric heating tests using SEM, Raman, and XRD, and compared with that before heating.

## 3. Results and Discussion

### 3.1. Morphology and Structure of the Electric Heating Membrane

**Figure 2:**
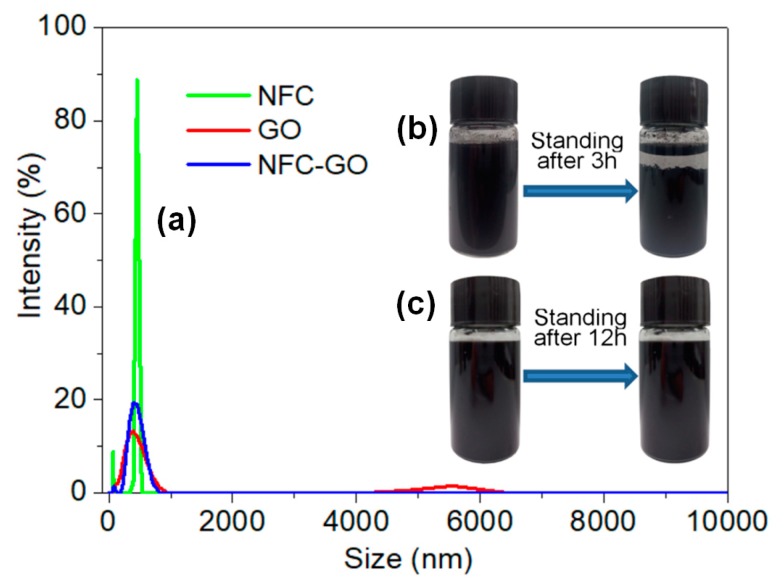
(**a**) Size distribution of the dispersions; (**b**) Standing stability of the graphene dispersion, concentration 0.25 mg·mL^−1^; (**c**) Standing stability of mixed dispersion with 50 wt.% graphene, GO:NFC = 1:1, 0.25 mg·mL^−1^.

Figure 2c displays the NFC-graphene-GO dispersion after standing for 12 h. The stability was better than that of the graphene dispersions (Figure 2b); notably, there was distinct stratification in the pure graphene sheet dispersion after standing for only 3 h (Figure 2b). The particle size distribution in Figure 2a also suggests that NFC and GO had been dispersed uniformly; the size distribution of the particles in NFC, GO, and NFC-GO dispersions was 396–459, 78–825, and 58–712 nm, respectively. Furthermore, the stability of these dispersion can be directly appraised by the zeta potential [22], which was measured as −46.8, −42.7, and −37.9 mV, respectively (Figure S3, Supplementary Materials). NFC and GO particles presented a negative charge in the water dispersion [23,24,25], giving the NFC-GO mixture negative charge that facilitated a stable homodispersion due to repulsion between particles with the same negative charge; of which, GO sheets exhibit negative zeta potential values mainly due to the presence of hydroxyl (O−) and carboxylic acid (COO−) groups on their surface [25]. Consequently, a stable NFC-graphene-GO dispersion was obtained. The electrostatic interaction induced by negative charges on the surface of the NFC and GO should have contributed to the formation of a network [23,24], which would include the graphene after it was ultrasonically mixed.

**Figure 3:**
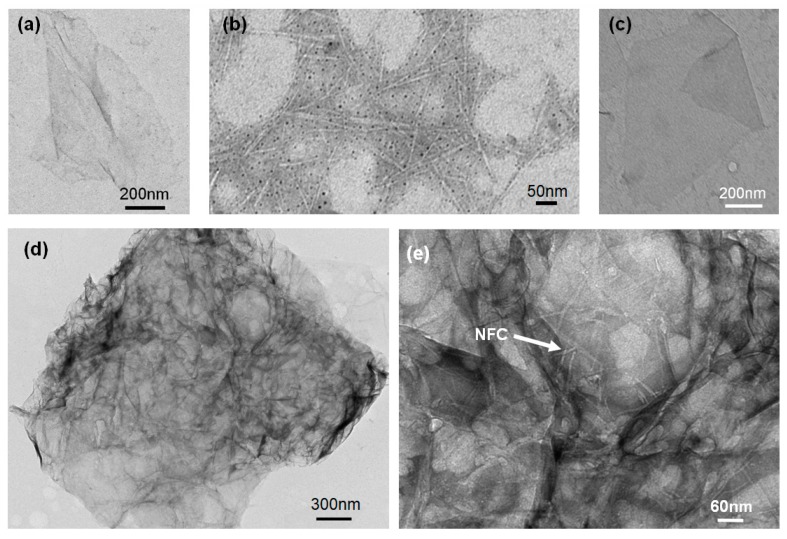
Transmission electron microscopy (TEM) images (100 kV) of (**a**) graphene; (**b**) nanofibrillated cellulose (NFC); (**c**) graphene oxide (GO); and (**d**,**e**) NFC-graphene-GO.

Figure 3a,c shows the morphology of graphene and GO sheet dispersions in distilled water; individual sheets can be observed with a folded appearance and high aspect ratio of diameter to thickness. This morphology would contribute toward the enhancement of the mechanical properties as well as the formation of a conductive network [15]. Figure 3b shows the whisker shape of the NFC, which had a diameter of about 20 nm. The length was less than the diameter of the GO and graphene sheet, as demonstrated by the cross lap network of interweaved nanofibrils among the sheets after ultrasonic mixing with GO and graphene (Figure 3d,e). Furthermore, a continuous large-area membrane was observed, which was embedded with skeletal NFC both among and joining the sheets (Figure 3e), indicating that a network constructed with NFC had formed in the system.

**Figure 4:**
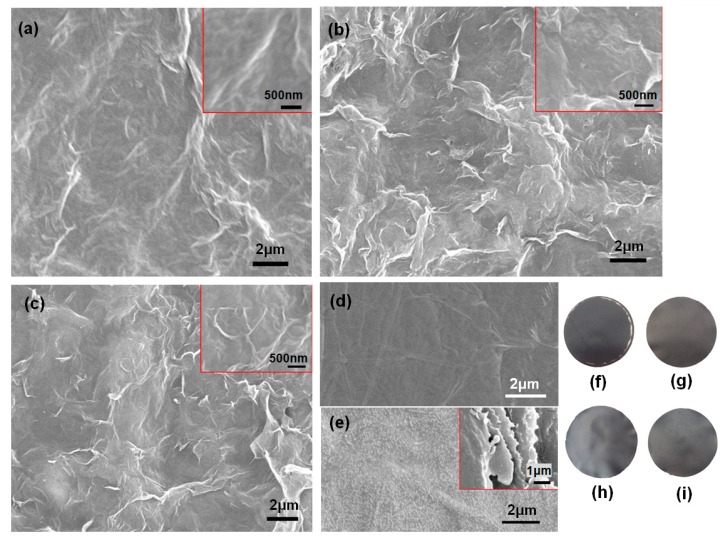
Scanning electron microscope (SEM) images of the surface morphology of composite membranes with (**a**) 20 wt.% graphene, GO:NFC = 1:1; (**b**) 50 wt.% graphene, GO:NFC = 1:9; (**c**) 50 wt.% graphene, GO:NFC = 1:1; (**d**) Pure GO membrane (16 g·m^−2^) formed by vacuum filtration; and (**e**) pure NFC membrane (16 g·m^−2^), also by vacuum filtration. A cross-section of the NFC membrane is inset in (**e**). Photographs of the membranes after drying: (**f**) 1 g·m^−2^ and (**g**) 8 g·m^−2^ grammage; (**h**) 30 and (**i**) 50 wt.% graphene.

**Figure 5:**
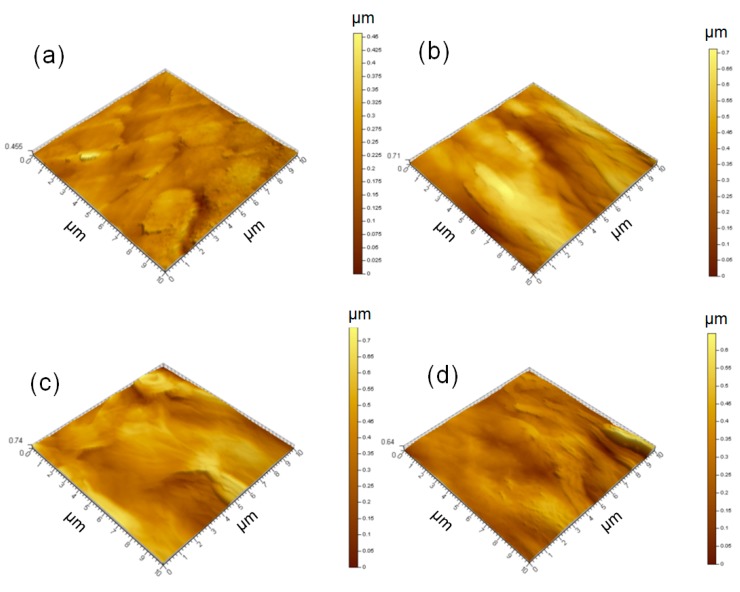
Atomic force microscopy (AFM) analysis on 16 g·m^−2^ membranes with (**a**) 30 or (**b**) 50 wt.% graphene; (**c**) 8 g·m^−2^ membrane with 50 wt.% graphene, all GO:NFC = 1:1; and (**d**) 16 g·m^−2^ membrane with 50 wt.% graphene, GO:NFC = 1:9.

SEM analysis on the resultant composite membranes exhibited a dense surface with a folded morphology, as demonstrated in Figure 4a–c, compared with the smoother surfaces of the pure GO and NFC membranes (Figure 4d,e). This further indicated the embedded structure in the membrane; i.e., that the graphene nanoparticles were dispersed uniformly in the 3D NFC matrix [26]. As the graphene content increased from 20 to 50 wt.%, the roughness noticeably increased. It could be attributed to the self-assembly of the graphene with a high aspect ratio of radius to thickness [26,27]. The NFC incorporated in the membrane could not be distinguished in Figure 4a–c due to the 2D graphene flakes on a large surface, while bacterial cellulose in published work [26,28] dispersed with the graphite nanoplatelets can been distinguished. Hence, the surface was not smooth, but showed a dense structure under high magnification (see the insets in Figure 4a–c). AFM analysis (Figure 5 and Figure S4, Supplementary Materials) confirmed that the surface was smoother when less graphene was added. Moreover, no distinct or agglomerated NFC was visible on the surface, which further suggested that the fibrillar NFC was uniformly dispersed, and had good compatibility with graphene and GO. In general, the membrane exhibited better surface quality as the amount of NFC and GO increased.

**Figure 6:**
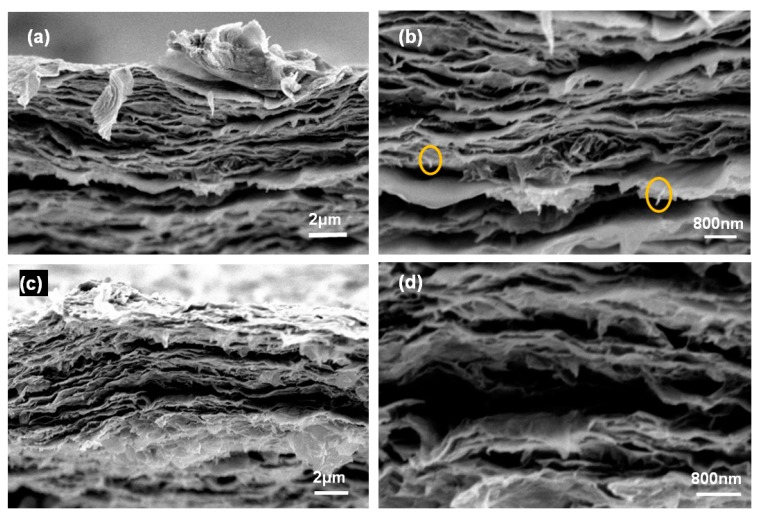
Torn cross-sections of membranes with (**a**,**b**) 30 and (**c**,**d**) 50 wt.% graphene.

Figure 6 showed a layered structure laminated by graphene sheets in the cross-section of the membranes, and similarly observed morphology have been also demonstrated in previous study [15,26,29]. This was attributed to the binding action of GO and NFC, which embedded among the graphene flakes, gluing them together. The fractured cross-sections were coarse, with a fibrillous torn morphology, indicating that there was strong adhesion among NFC, graphene, and GO in the membrane [15]. Consequently, it can be concluded that the NFC binder facilitated the construction of a 3D framework, and GO and graphene sheets were laminated in a disordered manner within the framework, which endowed the membrane with mechanical support.

**Figure 7:**
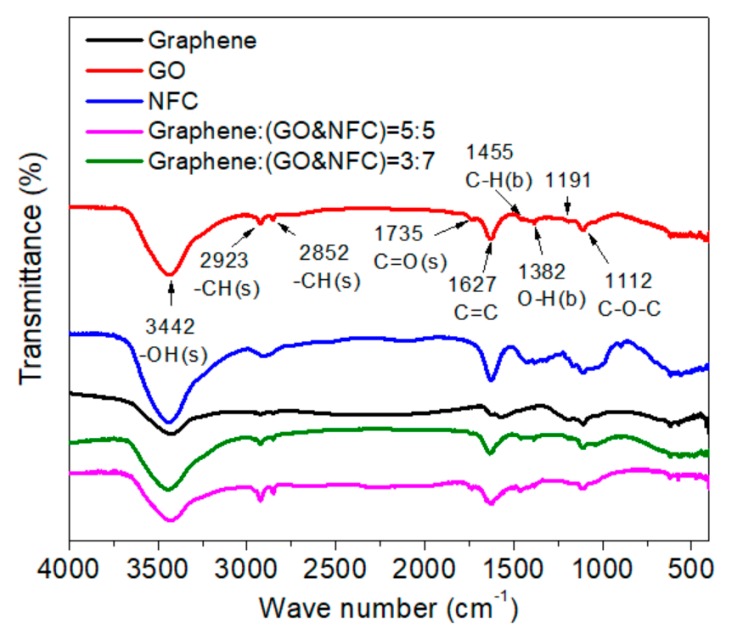
Fourier transform infrared (FT-IR) spectrum of composite membranes, GO, and NFC.

As shown in Figure 7, a broad and strong absorption peak centered around 3442 cm^−1^ is present, which corresponds to the stretching vibration of the –OH group in the GO and NFC samples [30,31]. The strength of this peak, as well as those of the C–O–C group located at 1191 and 1112 cm^−1^, was reduced in the presented composite membranes, which indicated that more intermolecular hydrogen bonds formed in the membrane with the –OH groups in GO and NFC [32]. Otherwise, the peaks at 2923 and 2852 cm^−1^, which correspond to the C–H group, were stronger for GO and the composite membrane than for NFC, whereas the peak at 1455 cm^−1^, which corresponds to the C–H deformation mode [31], exhibited more obviously for the graphene and composite membrane than for GO and NFC in Figure 7. Finally, C=C stretching vibration at 1627 cm^−1^ was present for GO, NFC, the composite membrane, and graphene.

### 3.2. Conductivity of the Electric Heating Membrane

**Figure 8:**
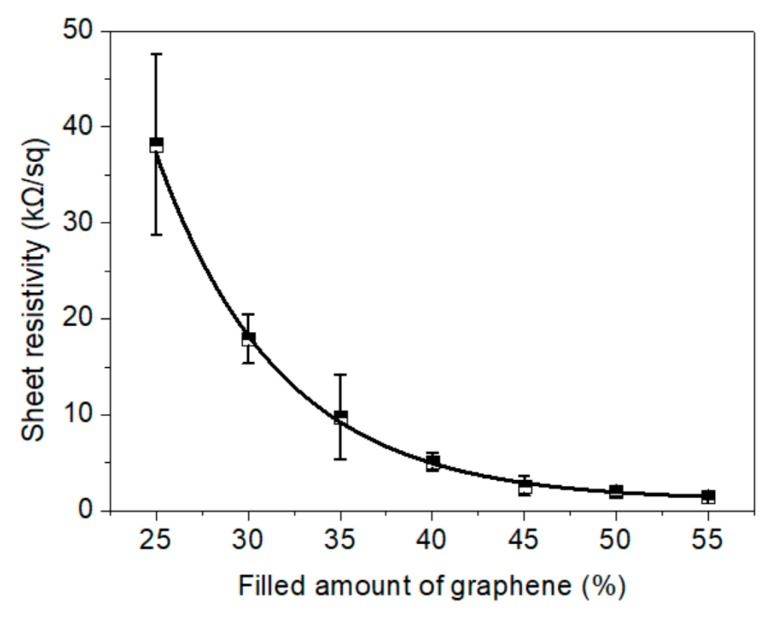
Sheet resistivity of the 16 g·m^−2^ membranes with different amounts of graphene.

A heater that utilizes the Joule heating effect should have appropriate conductivity, which is desirable for controlling the working power. As shown in Figure 8, as the amount of graphene increased, the sheet resistivity of the NFC–graphene–GO electric heating membrane exhibited a downtrend with a significant exponential function. The stabilized sheet resistivities of membranes with 40, 50, and 55 wt.% graphene were 5.13, 1.98, and 1.49 kΩ/sq, respectively. Furthermore, the standard deviation was less than that of membranes with ≤35 wt.% graphene.

**Figure 9:**
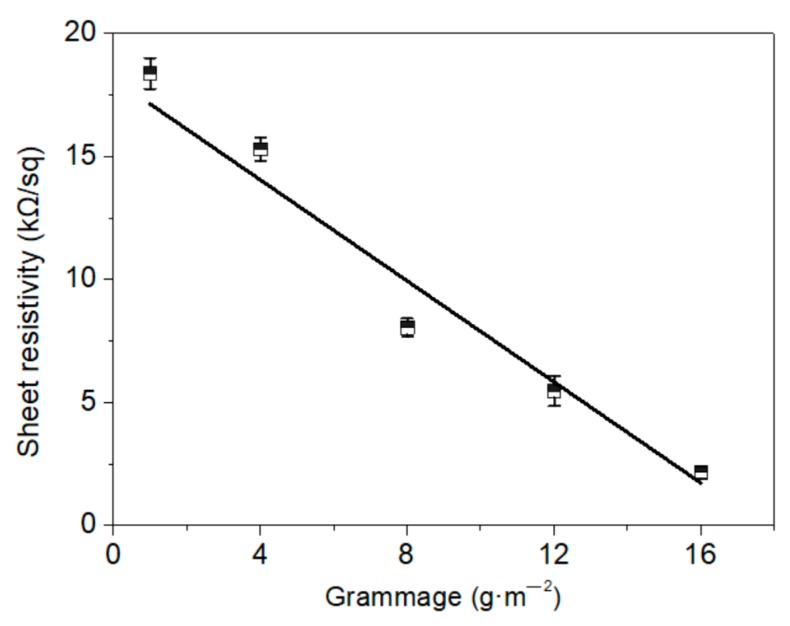
Sheet resistivity of the 50 wt.% graphene membranes with different grammages.

**Figure 10:**
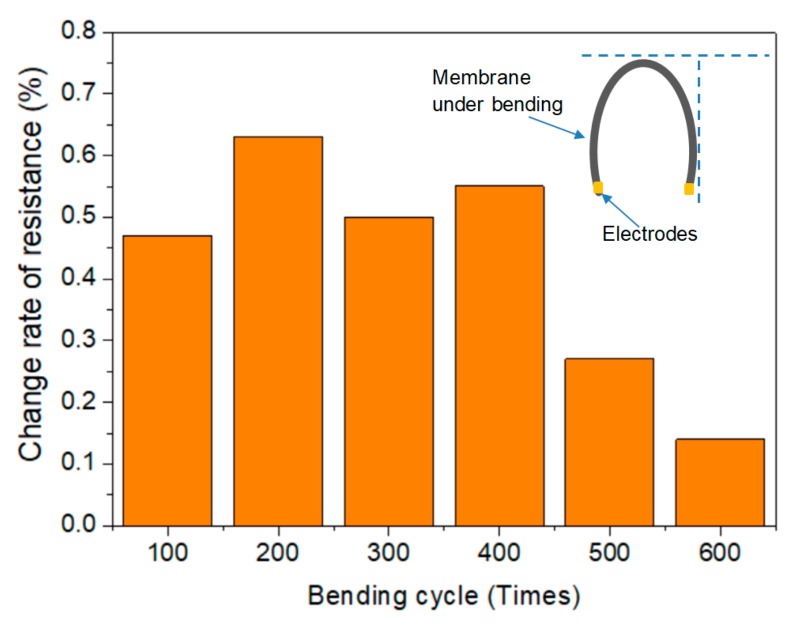
Stability of resistance between two electrodes of the 55 wt.% graphene membrane with grammage of 16 g·m^−2^ after different bending cycles.

The electrical properties can be controlled by changing the parameters of thickness, weight per unit area, or the composition. Much lower sheet resistivities of 1.75 to 9 Ω/sq have been demonstrated previously for membranes with the adjusted parameters [17,20,33]. Furtherly, the conductivity of membranes with various grammages (50 wt.% graphene) were investigated in this presented study. To the best of our knowledge, the relationship between grammage and conductivity has not been examined in existing reports, thus we aimed to consider saving material by producing a thinner membrane with suitable efficiency. A high linear relation between sheet resistivity and grammage was observed (Figure 9), with conductivity reducing significantly as the grammage decreased. The sheet resistivity of the lower grammage membranes of 1 and 4 g·m^−2^ reached 18.38 and 15.30 kΩ/sq, respectively; however, they also proved effective in the electric heating tests, as discussed below. In addition, Figure 10 shows that low change rate of resistance between two electrodes of the membrane after 100–600 times bending, in the range of 0.1 to 0.7%, which indicated its good stability and flexibility.

As shown in Figure S5 (Supplementary Materials), the graphene had an intact carbon structure even in the composite electric heating membrane. The ratios between the area of the D and G peaks (I_D_/I_G_) in the spectra were almost the same for graphene and the membrane (2.13 vs. 2.26), whereas both values were greater than that for GO (1.59) [34], indicating that NFC and GO disturbed the stacking behavior of graphene sheets to form the integrated structure. These findings were different from the results of Chen et al. [12], who demonstrated higher I_D_/I_G_ values with a RGO membrane, as pure GO sheets with abundant active groups stack more regularly and combine more compactly when cast and annealed by Joule heating. The position of the G peak of graphene and the membrane were located around 1587 and 1586 cm^−1^, respectively, while the D peak shifted from 1357 to 1361 cm^−1^. The full width at half maximum (FWHM) of the D peak increased from 207 to 221 cm^−1^, and that of the G peak increased slightly from 81 to 87 cm^−1^, meaning that integrity of the lamellar structure of graphene sheets embedded with NFC and GO had been slightly deteriorated. Slight variation of the phase structure of membranes with different amounts of graphene and GO were also found, as shown in Figure S6 (Supplementary Materials) and Table 1. Besides, both the 2D peak was weaker compared with the published RGO membrane [12], presenting a defective crystallized carbon structure, on account of the disturbance by NFC and GO embedded among the graphene sheets in the system. These findings indicate that the orientated structure in the membrane mainly depends on the stacking behavior of graphene and GO sheets under vacuum filtration, as well as crosslinking by the embedded NFC and GO binder.

**Figure 11:**
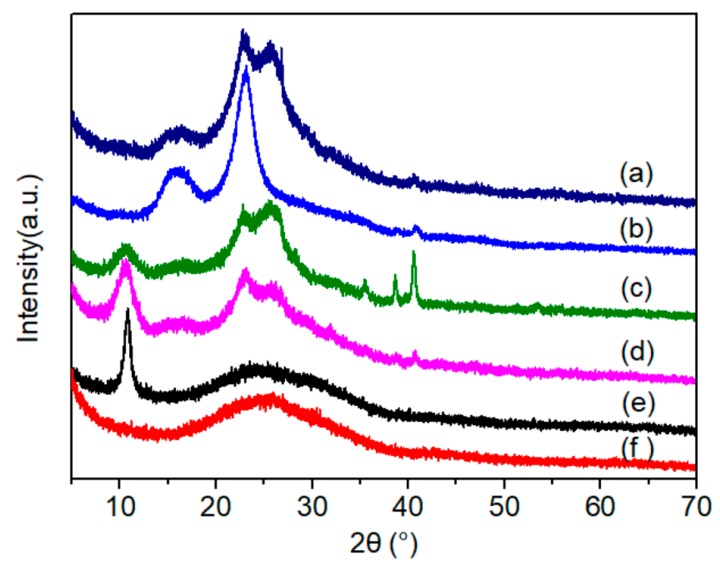
X-ray diffraction (XRD) patterns recorded for (**a**) 5 wt.% GO membrane (50 wt.% graphene); (**b**) NFC membrane; (**c**) 50 wt.% graphene membrane (GO:NFC = 1:1); (**d**) 30 wt.% graphene membrane (GO:NFC = 1:1); (**e**) GO membrane; and (**f**) graphene sheet.

The XRD pattern of the graphene sheet (Figure 11f) shows a gentle and wide reflection at around 2*θ* = 25.8°, indicating the well exfoliated state of the graphene layers compared with that in graphite [15,34,35,36]. This peak was noticeably narrower and stronger in the composite membranes (Figure 11a,b,d), implying a more compact stacked structure with laminated graphene layers cemented by GO and the NFC binder network. Furthermore, the peak was enhanced by decreasing the proportion of GO as well as by adding graphene. The diffraction peak near 2*θ* = 40.5° was strengthened as the amount of graphene was increased, and was prominent in the membrane with 50 wt.% graphene (Figure 11c), indicating that a much more regular structure formed among the stacked graphene layers [35]. This stacked structure is in accordance with the results of SEM and Raman analysis.

The characteristic reflections (2*θ* = 10.8°) of the GO membrane (Figure 11e) shows the lamellar spacing *d* as 0.819 nm according to Bragg’s diffraction formula [34,37]. In the composite membrane, after GO was crosslinked with NFC and graphene, the peak downshifted to 10.62° (*d* spacing = 0.832 nm) and became gentle, and was indistinguishable as the amount of GO decreased to 5 wt.%, due to the disruption caused by the added NFC and graphene. Hence the ordered layers of GO, which include oxygen-containing groups, are expected to be partly diverged [15,34].

### 3.3. Electric Heating Property

**Figure 12:**
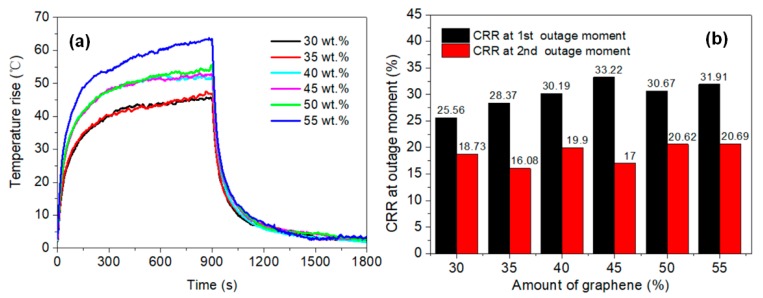
Electric heating performance (second heating test) of the 16 g·m^−2^ membranes with different amounts of graphene. (**a**) Temperature rise and (**b**) change rate of resistance (CRR) at the outage moment.

Figure 12a exhibits a smooth temperature rising process in the second test, while fluctuate process in the first test is shown in Figure S7 (Supplementary Materials), and the electrical parameters are shown in Table S1 (Supplementary Materials). As the graphene content increased, the balanced temperature rise in second test was greater under same power density of 1500 W·m^−2^, as shown in Figure 12a. The balanced temperature rise of the 55 wt.% graphene membrane exceeded that of the others. Moreover, the temperature rise increased by 15 °C more than in membranes with 30–35 wt.% graphene. The conductive network constructed by graphene sheets became more sophisticated with increased graphene content, and many overlaps and joints, which have higher contact resistance than graphene itself, formed among the graphene sheets. Thus, the main electric heating element was increased. This thermal resistance effect has been observed in many other graphene-based conductive composites [38,39,40]. For example, the resistance of a wooden electric heating composite was demonstrated to reduce after a current was applied, as well as the heat effect induced from Joule heating [41,42]. Figure 12b shows that, with increased graphene content, brittle holdback among the conductive network would multiply, which would open to a greater extent after power was applied, resulting in significantly enhanced conductivity. Consequently, the true power density in the heating process was higher under the same voltage, which was responsible for the observed differences in the balanced temperature rise. Moreover, the change rate of resistance (CRR) at the second outage moment presented minor variation among all the membranes, while a significant ascending trend was observed in the membranes with 30–45 wt.% graphene at the first outage moment. The CRR was stable in membranes with ≥45 wt.% graphene.

**Figure 13:**
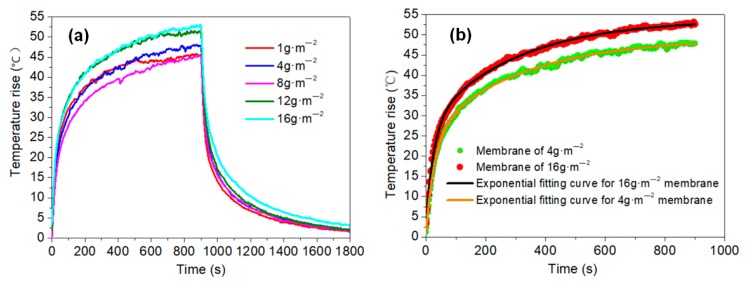
(**a**) Temperature rise of membranes with various grammages; (**b**) Exponential fitting of the 4 and 16 g·m^−2^ membranes.

The grammage difference between the 16 and 1 g·m^−2^ membranes was sizeable, with a large reduction in thickness, but their heating properties did not show any remarkable differences (Figure 13a), and a higher voltage can be applied to the thinner membrane. This phenomenon means that the membrane can be suitably made thinner and lighter, which is beneficial for the preparation of large surfaces. Generally, research into graphene-based membranes has been carried out with higher grammages, ranging from 25 to 250 g·m^−2^ [17,33], because thicker membranes show better conductivity. In which, the electrical parameters are shown in Table S2 (Supplementary Materials), and a fluctuant process in the first test is shown in Figure S8 (Supplementary Materials).

It has previously been indicated that the temperature rise in a plane electric heater will have an exponential trend with high correlation [42], and the membranes in the current study were no exception, as shown by the nonlinear curve fitting of the results in Figure 13b.

**Figure 14:**
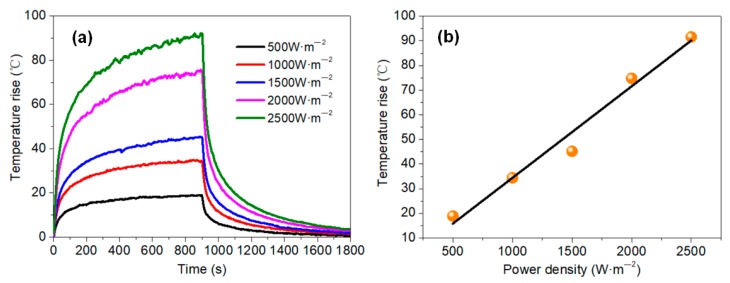
(**a**) Electric heating property of the 8 g·m^−2^ membrane with different power density; (**b**) Relation between temperature rise of the membrane and power density.

As shown in Figure 14, the temperature of the membrane rose quickly for the first 300 s, and then tended to stabilize. As the power density increased, the temperature rise of the membrane increased linearly, which can be illustrated from the fitting analysis results (*y* = −2.76 + 0.03716*x*; R^2^ = 0.98863; in which *y* is the temperature rise, and *x* is power density). Thus, a temperature rise of 90 °C can be achieved by applying 2500 W·m^−2^ power density (the electrical parameters are shown in Table S3, Supplementary Materials).

**Figure 15:**
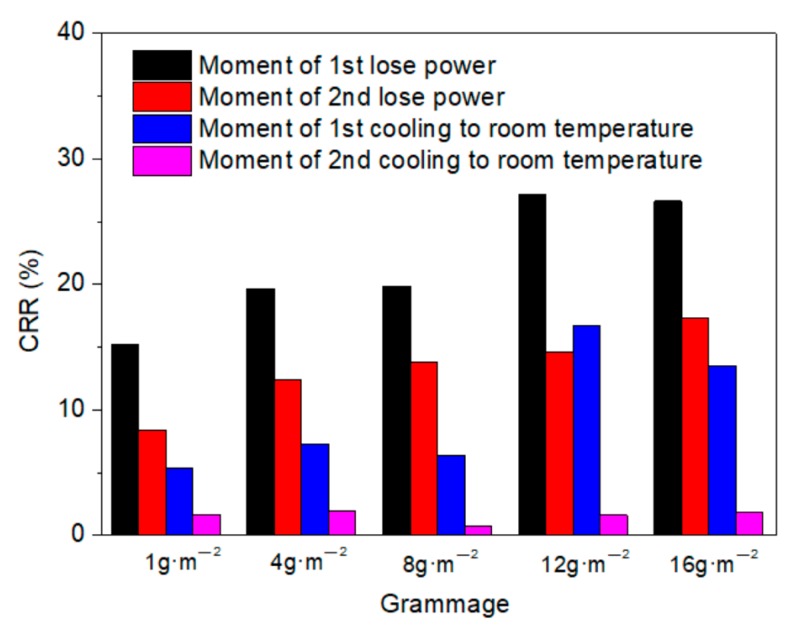
CRR of membranes with different grammages in the heating process.

The resistance stability in the power on process can be seen in Figure 15. For the 1 g·m^−2^ membrane, the CRR values at the moment the power was lost and after cooling to room temperature were 15.19% and 5.40% in first heating test, and 8.38% and 1.62% in the second test, respectively. As the grammage of the membrane increased to 16 g·m^−2^, the CRR values were 26.4% and 13.53% in the first test, and 17.34% and 1.83% in the second test, respectively. A larger CRR value was observed at the moment the power was lost, which decreased distinctly between the first and second tests. The CRR at the moment power was lost showed a linear relation with grammage, which contributes to the ability to control the real power when a current is applied. After cooling to room temperature, the CRR value declined more obviously, but there were only minor differences among the membranes with different grammages. This indicates that the stability of the conductive structure in the membrane increased after the first annealing test.

**Figure 16:**
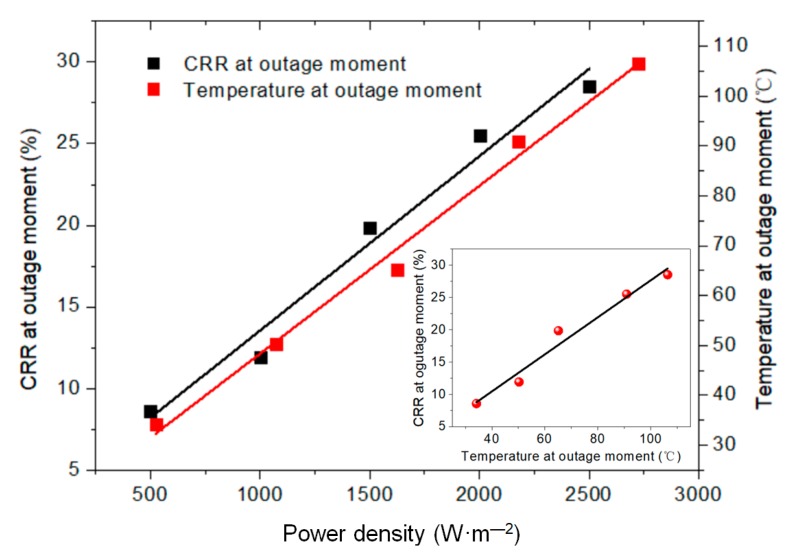
Relation among power density, CRR and temperature at outage moment for the 8 g·m^−2^ membrane.

Distinct linear relations were observed among the power density, CRR, and temperature at outage moment, as shown in Figure 16. The conductivity of the membrane increased linearly as temperature was increased by applying greater powers, which was supposed to be the negative temperature coefficient (NTC) effect [38,39,40].

### 3.4. Thermal Stability

**Figure 17:**
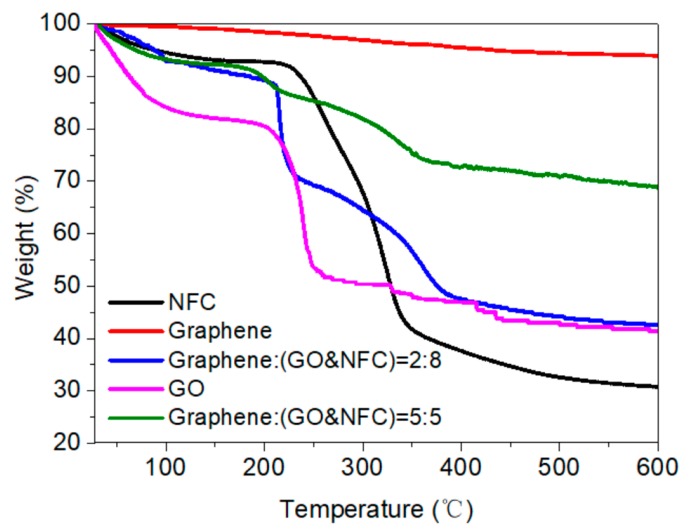
Thermogravimetric analysis (TGA) results for the graphene, NFC, GO, and composite membranes.

For application of the composite membrane as a heater, stable electric heating ability is essential, which requires thermal stability when only considering the heat effect. The excellent thermal stability of graphene has been studied in detail previously, leading to materials such as conductive nanographite-filled bacterial cellulose composites [26]. Figure 17 shows that the thermal property of the graphene in the current study was considerably stable, presenting a total mass loss of about 6% at 600 °C, whereas NFC and GO showed mass loss of 69% and 59%, respectively. It follows that an electric heating membrane with 20 wt.% graphene showed 57% mass loss, whereas those with 50 wt.% graphene showed only 31% mass loss. The mass loss would be decreased by increasing the amount of graphene, as found in nanographite-filled bacterial cellulose composites [26].

**Figure 18:**
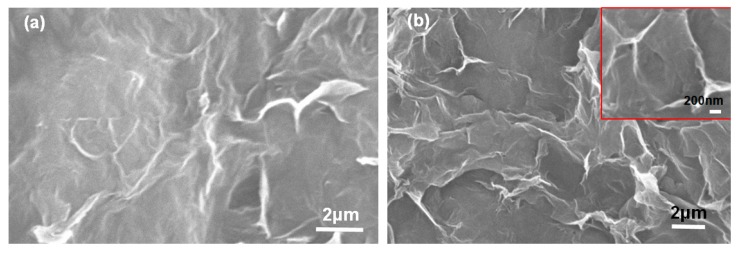
Surface morphology of the membrane with 50 wt.% graphene (**a**) before and (**b**) after the electric heating test.

**Figure 19:**
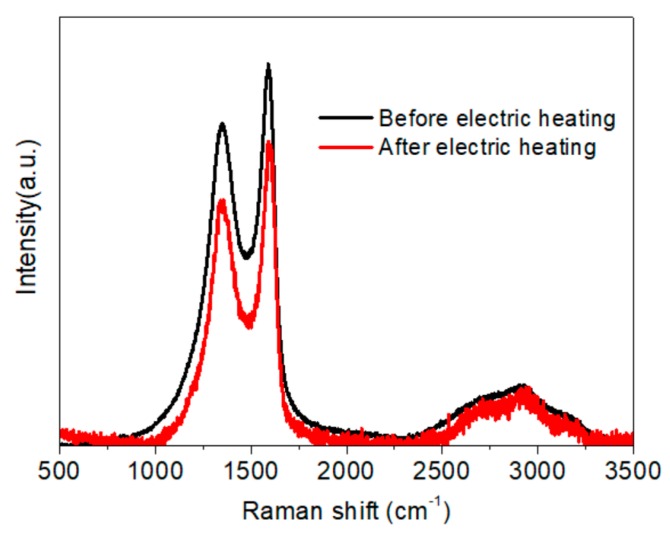
Raman spectra for the 16 g·m^−2^ membrane with 50 wt.% graphene before and after the electric heating test.

After the electric heating test, the surface stacking density of the membrane increased, as shown in Figure 18, because the irregularities of the total structure were reduced [12]. This was substantiated by the results of Raman analysis shown in Figure 19. Moreover, the skeletal structure became more distinct after the irregularities were reduced, with a more compact arrangement among the graphene and GO sheets in the 3D system.

**Figure 20:**
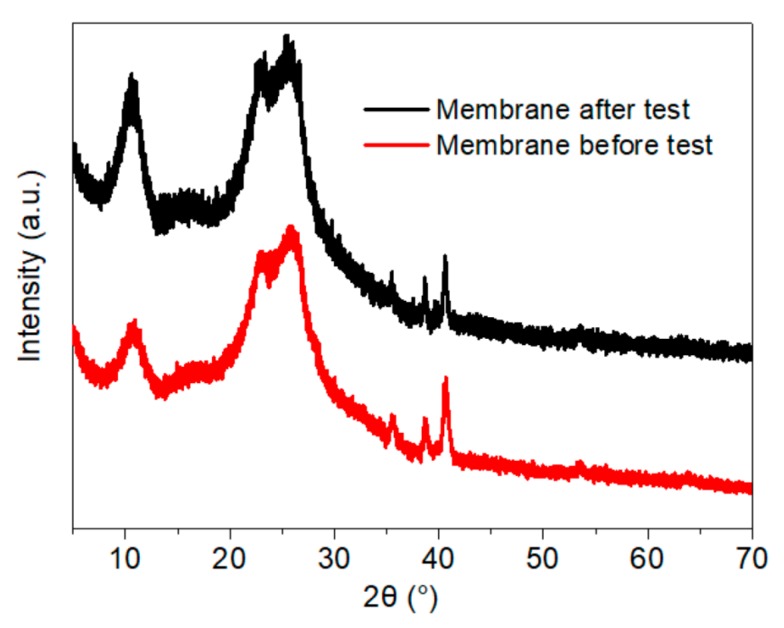
XRD spectra for the 16 g·m^−2^ membrane with 50 wt.% graphene before and after the electric heating test.

Under the function of Joule heating induced by an applied electric current, the phase structure of graphene-based composites has previously been shown to be improved to some degree [4,12]. As shown from the results of Raman analysis in Figure 19, the structural regularity of the membrane was enhanced after two electric heating tests, according to the ratio between the area of the D and G peaks (I_D_/I_G_) in the spectrum, which decreased from 2.21 to 1.84. Likewise, the I_2D_/I_G_ ratio declined slightly from 0.67 to 0.59, and the peak position shifted to a higher wavenumber. The FWHM of the D, G, and 2D peaks all decreased, from 216 to 188 cm^−1^, 86 to 81 cm^−1^, and 398 to 336 cm^−1^, respectively. The change in I_D_/I_G_ values differed from those in published works; however, while previous studies focused on neat RGO reduced by Joule heat with high power [4,12], we used medium and low power Joule heat in this research for its heating application. Furthermore, the NFC used as a binder for endowing the membrane with better adhesion and mechanical performance in this work influenced the lamination of the graphene and GO sheets during vacuum filtration. The diffraction peak near 2*θ* = 10.7° in the XRD pattern (Figure 20) became stronger and sharper after the electric heating test, which also indicated the formation of a more regular structure stacked with graphene sheets.

## 4. Conclusions

1. NFC, graphene, and GO were dispersed uniformly and adequately in the aqueous system. The negative charge on the surface of NFC and GO, as well as their high length–diameter and radius–thickness ratio, meant that they formed a crosslinked structure, facilitating the dispersion of graphene and stability of the system. NFC and GO act as a binder, and form bonds between two sheets. Furthermore, the skeletal NFC was lapped across the membrane. The cross-section of the membrane revealed a layered and rough structure, while the surface was smooth and compact. These results indicate that a 3D network had formed in the membrane, generating a certain mechanical and conductive property.
2. Graphene sheets with a high aspect ratio contributed toward the enhanced conductivity of the membrane. The sheet resistivity of the electric heating membrane exhibited a downtrend with a significant exponential function as the amount of graphene increased, while there was a high linear relation between the sheet resistivity and grammage. This implies that the conductivity can be controlled for a specific target power and supply voltage, and that NFC and GO disturbed the stacking behavior of the graphene sheet to form the integrated structure.
3. The temperature rise on the membrane surface was elevated when higher amounts of graphene were added, and increased linearly with the applied power density. Both Joule heating and the electric current led to the decline of membrane resistance. The temperature rose quickly for the initial 300 s and then stabilized, presenting an exponential function trend with high correlation. The heating properties of the membranes did not show significant differences: even when the grammage was decreased from 16 to 1 g·m^−2^, the membranes showed a stabilized temperature rise of about 52 and 45 °C, respectively, under the same power density of 1500 W·m^−2^.
4. The thermogravimetric analysis (TGA) results indicated that thermal stability of the membrane was improved due to the crosslinking of NFC and GO. The regularity of the membrane structure was elevated after two heating tests, with regard to the CRR at the moment power was lost and after cooling. The CRR showed suitable relationships with the grammage and graphene content, which will contribute to the control over the actual power in use. However, before further application, additional mechanisms and technologies must be investigated, such as the variation of phase structure induced by Joule heating with a wider range of power and longer working time, the relation between phase structure and thermal radiation, and key technologies for large scale printing or coating.

## Figures and Tables

**Table 1:** Raman peak positions and FWHM, and intensity ratios of I_D_/I_G_ and I_2D_/I_G_.

Samples *	D Peak		2D Peak		G Peak		I_D_/I_G_	I_2D_/I_G_
	Peak Positions (cm^−1^)	FWHM of Peak (cm^−1^)	Peak Positions (cm^−1^)	FWHM of Peak (cm^−1^)	Peak Positions (cm^−1^)	FWHM of Peak (cm^−1^)		
(a)	1360.21	204.64	3861.65	387.20	1587.76	87.28	2.03	0.66
(b)	1360.54	195.18	2854.45	409.73	1586.30	89.14	1.88	0.70
(c)	1360.86	221.32	2821.22	437.79	1588.42	86.81	2.26	0.78
(d)	1361.41	223.24	2826.91	430.56	1588.27	85.91	2.27	0.77
(e)	1362.30	219.49	2835.86	434.15	1589.08	86.15	2.19	0.83
(f)	1361.75	224.48	2807.36	473.43	1589.96	85.85	2.27	0.88

FWHM: full width at half maximum; * Samples with GO:NFC = 1:1 and (a) 20, (b) 30, and (c) 50 wt.% graphene, and with 50 wt.% graphene and (d) 5, (e) 15, and (f) 30 wt.% GO.

## Supplementary Materials

The following are available online at http://www.mdpi.com/1996-1944/11/9/1727/s1, Equation (S1) and (S2): calculation for voltage (U) applied on two electrodes, Figure S1: specific position for the test of sheet resistance on the membranes, Figure S2: installation of electrodes and temperature senor on the membrane, Figure S3: zeta potential distribution of (a) NFC, (b) GO, and (c) NFC–GO dispersions, Figure S4: two-dimension AFM analysis on the membranes, Figure S5: Raman spectra of graphene, GO, and the membrane, Figure S6: Raman spectra of membranes with GO:NFC = 1:1 and (a) 20, (b) 30, and (c) 50 wt.% graphene, and with 50 wt.% graphene and (d) 5, (e) 15, and (f) 30 wt.% GO, Table S1: electrical parameters in the heating test for the membrane with various amount of graphene with grammage of 16 g·m^−2^ and the ratio between GO and NFC as 1:1 (under the power density of 1500 W·m^−2^), Figure S7: temperature rise on the membrane with different amount of graphene in the first heating test under the power density of 1500 W·m^−2^, Table S2: electrical parameters in the heating test for the membrane with various grammage under the power density of 1500 W·m^−2^, Figure S8: temperature rise on the membrane with various grammage in the first heating test under the power density of 1500 W·m^−2^, Table S3: electrical parameters in the heating test of the membrane (grammage of 8 g·m^−2^) inputted with different power density.
