# Supplementary material for: Thin Electric Heating Membrane Constructed with a Three-Dimensional Nanofibrillated Cellulose–Graphene–Graphene Oxide System
Source: Materials (Basel). 2018 Sep 14;11(9):1727. doi: 10.3390/ma11091727 (PMC6163438; doi:10.3390/ma11091727)
Supplement: Supplementary file 1 [file materials-11-01727-s001.pdf]

Supplementary Materials

Thin Electric Heating Membrane Constructed with a Three-Dimensional Nanofibrillated Cellulose—Graphene—Graphene Oxide System

Chuang Shao, Zhenyu Zhu, Chuwang Su, Sheng Yang, Quanping Yuan*

School of Resources, Environment and Materials, Guangxi University, Nanning 530004, China; icshao@163.com (C.S.); zhenyzhu@163.com (Z.Z.); glscw58@163.com (C.S.); yangs0825@163.com (S.Y.);

*Correspondence: yuanquanping@gxu.edu.cn; Tel.: +86-0771-3232-200

Voltage (U) applied on two electrodes under a given power density and resistance between the two electrodes on the electric heating membrane was calculated according to the equation (S1) and (S2).

$$U=(R*P)^{1/2} \qquad \text{(S1)}$$

$$P=p*s \qquad \text{(S2)}$$

In which, U, R, P, p, s are voltage (V) applied on two electrodes of the membrane, resistance (Ω) between two electrodes, total power (W) applied on the electrodes, power density (W·m^{-2}), effective heating surface (m^2, 20mm×30mm), respectively.

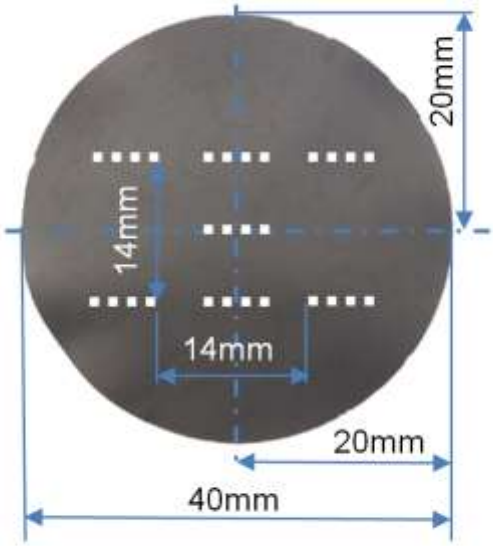

Figure S1. Specific position for the test of sheet resistance on the membranes.

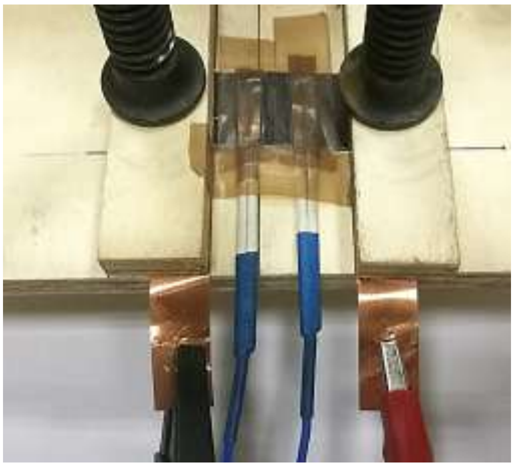

Figure S2. Installation of electrodes and temperature senor on the membrane. In which, heat and electrically resisting tape was used for the installation of temperature senor.

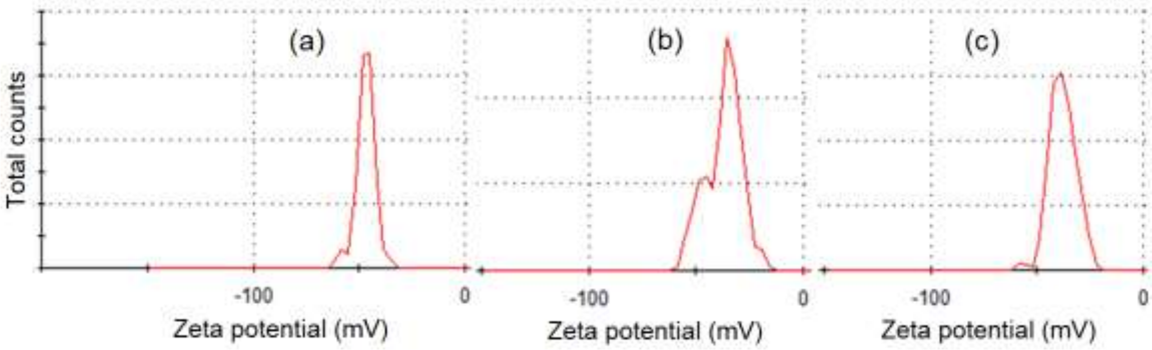

Figure S3. Zeta potential distribution of (**a**) NFC, (**b**) GO, and (**c**) NFC–GO dispersions.

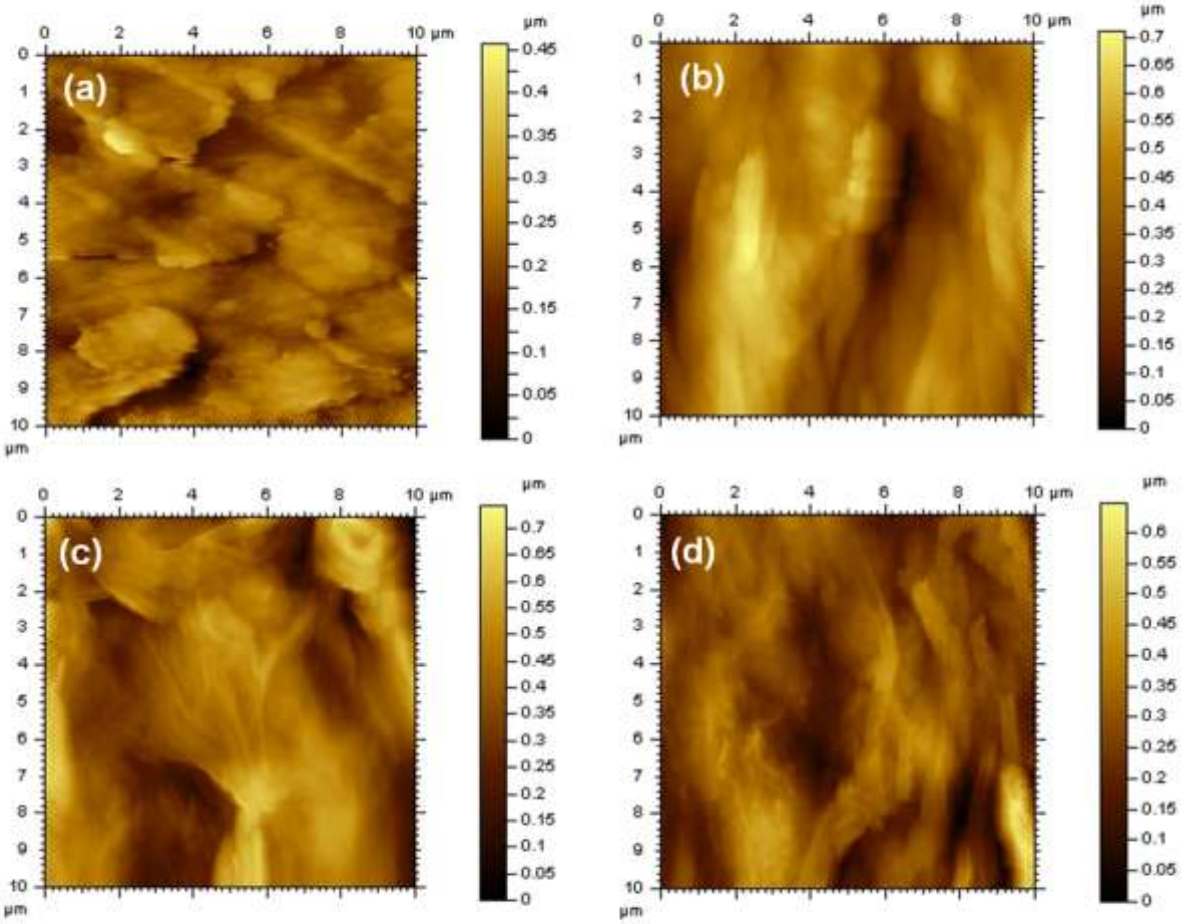

Figure S4. Two-dimension AFM analysis on 16 g •m^{-2} membranes with (a) 30 or (b) 50 wt.% graphene, (c) 8 g·m^{-2} membrane with 50 wt.% graphene, all GO:NFC = 1:1, and (d) 16 g·m^{-2} membrane with 50 wt.% graphene, GO:NFC = 1:9.

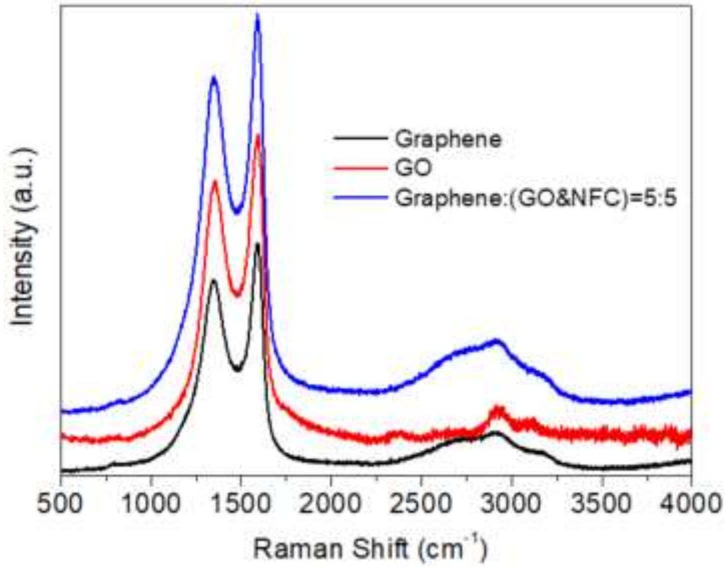

Figure S5. Raman spectra of graphene, GO, and the membrane.

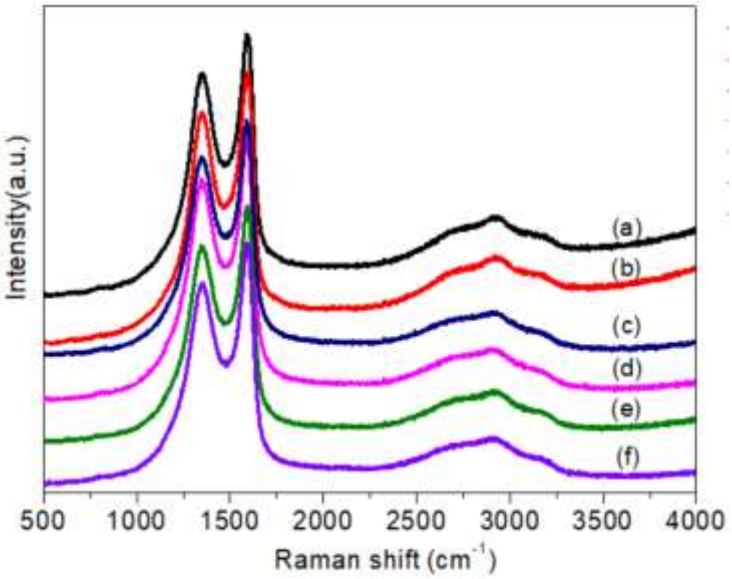

Figure S6. Raman spectra of membranes with GO:NFC = 1:1 and (**a**) 20, (**b**) 30, and (**c**) 50 wt.% graphene, and with 50 wt.% graphene and (**d**) 5, (**e**) 15, and (**f**) 30 wt.% GO.

Table S1. Electrical parameters in the heating test for the membrane with various amount of graphene with grammage of 16 g·m^{-2} and the ratio between GO and NFC as 1:1 (under the power density of 1500 W·m^{-2}).

Membrane with different amount of graphene	First heating test parameters		Second heating test parameters	
	R_1 (KΩ)	U_1 （V）	R_2 (KΩ)	U_2 (V)
30 wt.%	23.550	146	22.260	142
35 wt.%	14.610	115	12.995	108
40 wt.%	7.535	82	6.685	78
45 wt.%	5.580	71	4.765	65
50 wt.%	3.750	58	3.274	54
55 wt.%	2.326	46	2.004	43

R_1: resistance between two electrodes of the membrane before the first electric heating test; R_2: resistance between two electrodes of the membrane before the second electric heating test. U_1: voltage applied in the first test; U_2: voltage applied in the second test.

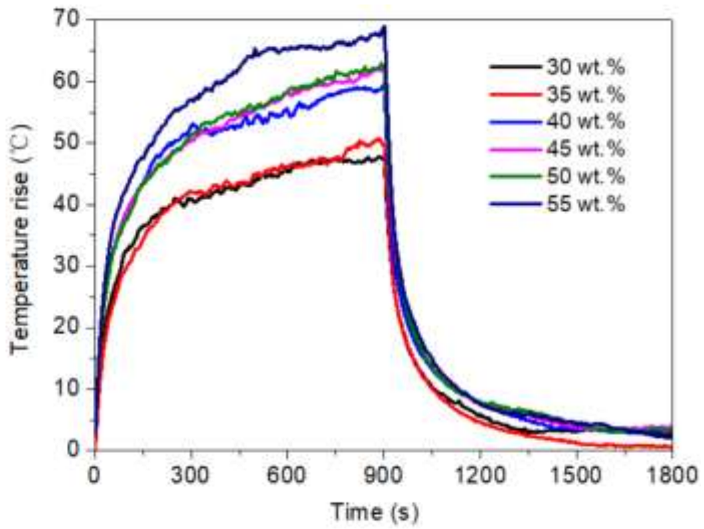

Figure S7. Temperature rise on the membrane with different amount of graphene in the first heating test under the power density of 1500 W·m^{-2}.

Table S2. Electrical parameters in the heating test for the membrane with various grammages under the power density of 1500 W·m^{-2}.

Membrane with various grammages (g·m^{-2})	First heating test parameters		Second heating test parameters	
	R_1 (KΩ)	U_1（V）	R_2 (KΩ)	U_2 (V)
1	20.925	137	20.000	134
4	16.545	122	15.360	118
8	6.215	75	5.920	73
12	4.420	63	3.729	58
16	3.667	57	3.181	54

R_1: resistance between two electrodes of the membrane before the first electric heating test; R_2: resistance between two electrodes of the membrane before the second electric heating test. U_1: voltage applied in the first test; U_2: voltage applied in the second test.

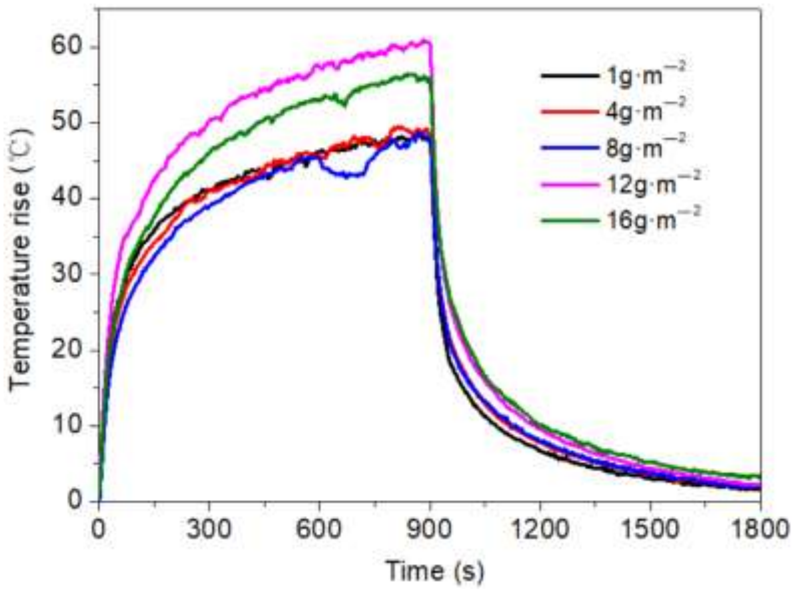

Figure S8. Temperature rise on the membrane with various grammages in the first heating test under the power density of 1500 W·m^{-2}.

Table S3. Electrical parameters in the heating test of the membrane (grammage of 8 $g·m^{-2}$) inputted with different power density.

**Power density ($W·m^{-2}$)**	**Heating test parameters**	
	**R (KΩ)**	**U (V)**
500	6.400	44
1000	6.290	61
1500	5.920	73
2000	6.150	86
2500	5.765	93

R: resistance between two electrodes of the membrane before the electric heating test; U: voltage applied in the test.
